# Virtual Emergency Medicine Clerkship Curriculum during the COVID-19 Pandemic: Development, Application, and Outcomes

**DOI:** 10.5811/westjem.2021.2.48430

**Published:** 2021-04-28

**Authors:** Kathryn E. Redinger, Jeffrey D. Greene

**Affiliations:** * Western Michigan University Homer Stryker M.D. School of Medicine, Department of Emergency Medicine, Kalamazoo, Michigan; † Western Michigan University Homer Stryker M.D. School of Medicine, Department of Medical Education, Kalamazoo, Michigan

## Abstract

The COVID-19 pandemic has been a significant catalyst for change in medical education and clinical care. The traditional model of bedside clinical teaching in required advanced clerkships was upended on March 17, 2020, when the Association of American Medical Colleges recommended removing medical students from direct patient care to prevent further spread of the disease and also to help conserve scarce personal protective equipment (PPE). This created unique challenges for delivering a robust, advanced emergency medicine (EM) clerkship since the emergency department is ground zero for the undifferentiated and potentially infected patient and has high demand for PPE. Here, we describe the development, application, and program evaluation of an online-based, virtual advanced EM curriculum developed rapidly in response to the COVID-19 pandemic.

Starting March 23, 2020, we began rotating fourth-year medical students through a four-week rotation. We completed a total of four virtual clerkship experiences comprised of 56 students through July 27, 2020. Through analysis of the students’ performance on a national standardized EM shelf exam, students participating in this virtual clerkship demonstrated a fund of knowledge that was not significantly different from that of their peers who completed a traditional clerkship in the specialty prior to the pandemic interruptions. Additionally, the critical review of the traditional course created the opportunity to make improvements and enrich the medical student educational experience in a virtual environment and upon resumption of the traditional course when students returned to the in-person environment. The resources provided for those interested in adopting our pedagogical approach include a course syllabus, calendar, and learner summative assessment.

## BACKGROUND

**Figure 1:**
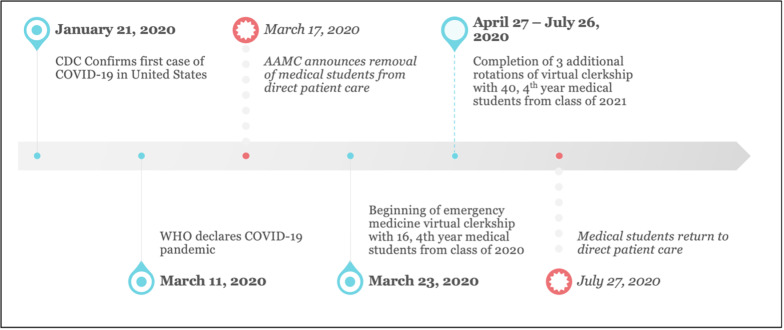
Timeline of the development and implementation of a virtual emergency medicine clerkship during the COVID-19 pandemic.^3^

In 2006 Cooke and her colleagues observed in a *New England Journal of Medicine* review article that “Medical Education seems to be in a perpetual state of unrest.”^1^ Nationally the need to develop a virtual clerkship model became necessary when the Association of American Medical Colleges (AAMC) issued a statement strongly discouraging students from direct patient care in light of the COVID-19 pandemic, and extended stay-at-home orders were implemented in states across the country. This removed opportunities for bedside teaching in the course of direct patient care but also eliminated the ability for students and faculty to meet in classroom and simulation environments. Although the modern era has seen a large increase in technological innovation and online-based learning modules, up until now those advances have always served to augment traditional bedside training, not supplant it (Figure 1).^2^

Our institution has a required fourth-year emergency medicine (EM) curriculum for all students. Sixteen of the fourth-year students from the Class of 2020 who were previously enrolled in the March 23 rotation were switched to the virtual learning environment to meet graduation requirements. In the spring, fourth-year students of the Class of 2021 were given the option of completing the virtual curriculum to fulfill their EM requirement or take their EM clerkship in the fall/winter in the traditional clinical model. Forty students elected to enroll in the virtual EM curriculum from April 27–July 26, 2020, representing 49% of the Class of 2021.

Students were able to return to the clinical environment in late summer; however, we still experienced periodic interruptions in medical education due to the need for quarantine and isolation among our learners. Moreover, in January 2021 the AAMC recommended that medical students not be involved in the direct care of known or suspected COVID-19 patients. For those students able to participate in direct patient care the undifferentiated nature of acute emergency patients, and the wide variability of presenting complaints in active COVID-19 disease, has limited the number of patients the medical students could see and the number of procedures they were allowed to participate in despite their return.

Although emergency departments (ED) provide robust learning opportunities for medical students through the acute, undifferentiated patient, evidence suggests that student exposure to recommended curriculum presentations and procedures was limited even before COVID-19. In a study published in 2014, case logs from 130 students at three institutions were reviewed. Only 15.4% of students saw 10 required conditions during their rotation, although 76.9% saw at least eight.^4^ This finding provides evidence that even in the traditional bedside model, advanced clerkship curriculums are lacking key educational components since certain conditions are far less likely than others to be encountered.

The lack of clinical exposure available to students was compounded by a general decrease in ED patient volumes nationally. According to the National Syndromic Surveillance Program, ED visits declined 42% during the early COVID-19 pandemic.^5^ Volumes continued to languish through the summer months, and an analysis from TransUnion Healthcare found ED visits were down 25% through August 2020.^6^ As students return to patient care in EDs, educators must be prepared for the possibility that volumes will not be sufficient to ensure a robust educational experience without supplemental material taught in a nonclinical setting.

**Population Health Research Capsule**

What do we already know about this issue?

*Medical students were removed from the clinical learning environment in response to the COVID-19 pandemic.*

What was the research question?

*How are virtual students’ educational outcomes different from students in clerkships with direct patient contact?*

What was the major finding of the study?

*Virtual students’ exam scores were not significantly different from peers’ scores in the in-person setting.*

How does this improve population health?

*Beyond the COVID-19 pandemic interruptions, this virtual curriculum is useful in situations where students have limited access to direct patient care.*

Having a prepared contingency plan for an online-based curriculum for students who are experiencing interruptions in their education due to quarantine, isolation, or other unforeseen events or absences not related to COVID-19 is a beneficial resource. A bank of chief complaint-based activities and resources can help augment an in-person ED clerkship if there is a specific, patient encounter deficiency identified by the student or educator. This course as it is designed in its entirety also has utility in a post-pandemic world as an elective offering for both EM and non EM-bound students interested in building the medical knowledge base of EM in self-directed study. This study was granted exception status by the Western Michigan University Homer Stryker M.D. School of Medicine institutional review board.

## OBJECTIVES

At the outset, the virtual clerkship curriculum was designed to capture as many of the institutional educational objectives contained within the live clerkship as possible to ensure that our students were minimally disadvantaged by the change in content delivery. The course was to remain academically rigorous, so that COVID-19 did not become an excuse for students to receive a watered-down version of the curriculum or progress without an EM experience altogether. Table 1 summarizes the key changes in curriculum design and assessment methodology for each learning objective from an in-person clerkship to a virtual one.

## CURRICULAR DESIGN

Using Kern’s six-step model for curriculum development in medical education, we developed a four-week course for the fourth-year medical student that combined independent, self-directed learning with live, synchronous team-based discussions that served as a replacement for a hands-on, direct patient encounter-oriented EM clerkship.^7^ Due to time constraints, we were not able to perform a formal targeted needs assessment of the students or to survey academic faculty. Rather, conversations among the academic faculty in the days following the removal of students from the clinical environment identified the desire for team-based discussions focused on the most frequently encountered chief complaints and strategies to increase student interaction and engagement.

Self-directed, lifelong learning is an essential component to the professional development of physicians.^8^ A completely asynchronous course could serve adequately to deliver the content. However, by maintaining the social structure of the clerkship environment and moving to regular, live, synchronous, team-based discussions, students were supported and empowered in a more engaging learning environment. In *Understanding Medical Education*, Kaufman and Mann write, “Learning occurs not only individually, but in collaboration with others.”^8^ Collaborative strategies employed in team-based sessions included the following: role-playing with debriefing in an oral board exam format; case discussions; traditional lectures; and question- and-answer sessions to help students with challenging material from their self-directed learning.

The course was organized by the 12 most common chief complaints and procedures students would encounter in the clinical setting. Woven throughout the course were sessions in radiology and electrocardiogram (ECG) interpretation and supplemental learning from textbooks, primary journal articles, podcasts, online board review, and Free Open Access Medicine blog posts. In choosing these particular resources, the aim was to recreate the bedside teaching of preceptors by incorporating review of ECGs and imaging studies and opportunities to hear from experts in the field clinical pearls, personal experiences, primary literature, and high-yield questions they might have been asked on shift. The students completed a daily quiz to reinforce topics and in preparation for the National Board of Medical Examiners (NBME) shelf exam. Each student was also asked to prepare a case presentation (Appendix 1: Course Syllabus, Appendix 2: Course Calendar).

### Resources

We found that there were advantages and disadvantages of the resources we used in building our curriculum (Table 2). The transition of the medical school to Microsoft Teams via our Office 365 platform (Microsoft Corporation, Redmond, WA) was seamless and offered both security and functionality in classroom management advantages over other video- and audio-conferencing software. Using the software, virtual classrooms were built in the Microsoft Teams platform, which allowed one place for video conferences, news feed with chat functions, class assignments, daily quizzes, and grade book.

We also used curated content built around simulated patient encounters employing Online MedEd Case X (Online MedEd, Austin, TX) videos and Emergency Medicine Reviews and Perspectives (EM:RAP) (EM:RAP, Inc., Burbank, CA) podcast audio of EM patients and relevant cases.^8^ We aimed to find resources with opportunities to observe the sights and sounds of the ED using multimedia formats as a surrogate for the obvious deficiency of patient-student interaction. Online MedEd Case X provided video of real patients captured by emergency medical services or clinical staff of the patient interview. The main limitation of this particular resource was that it was not EM specific. As a result, encounters most relevant to the ED were selected from their available cases for internal medicine, obstetrics/gynecology, surgery, pediatrics, and psychiatry. Audio content, especially the C3 series from EM:RAP, was used because it provided robust audio content specific to EM, including patient interviews.^9^,^10^

### Challenges

The most obvious challenge was the lack of immersive experiential learning (Table 3). Although the formal curriculum and educational objectives remained unchanged, with adaptations only to content delivery and assessment, components of the informal curriculum that come from the apprenticeship in the direct clinical environment could not be duplicated. This particular challenge was congruent with limitations described during a novel urologic medical student virtual subinternship developed in response to the COVID-19 pandemic.^11^

During weekly interactive, didactic Teams meetings and also during mock oral board-style cases, instructors assumed the role of patients to re-create opportunities for patient-student interviews. These encounters were well received and provided much needed feedback to the learner about their interviewing skills. Unfortunately, lack of time and available instructors was a significant limitation to providing more opportunities like this. Use of procedural skills training, simulation, and standardized patient would be an obvious surrogate to help mitigate some of this. However, our simulation center was closed due to state-mandated stay-at-home orders. Additions of these types of events for future iterations of this course would be beneficial.

Additional challenges included technical problems common to online meetings such as poor connectivity, background noise, or unintentional unmuted distractions. Dost and colleagues reported their survey of over 2500 United Kingdom medical students’ perceptions of online medical instruction during the COVID-19 pandemic and found that the most commonly cited student barriers to learning in the virtual environment were family distraction (26.76%) and poor internet connection (21.53%).^12^ Recognition of perceived student barriers to learning may help to identify students vulnerable to the challenges of the virtual environment.

Not exclusive to EM, or even to medical education broadly, fostering participation in the virtual setting is difficult. Success in this environment is dependent on active participation and engagement throughout the course.^13^ Our instructors reported that teaching in an online environment can feel lonely if students are participating by calling into sessions but not using the video technology or other chat features. Our students also reported feelings of isolation from their peers in this environment. Incorporating opportunities for group discussion or participation through games, polls, or small-group breakouts may help to mitigate this.^13^,^14^ Clear expectations for attendance, participation, and communication are essential and may be incorporated into institutional policy.

## IMPACT / EFFECTIVENESS

Evaluation of students in the prior, in-person clerkship used an honors, high pass, pass, and fail grading scale based on patient encounter presentations, professionalism, and the NBME shelf exam. We simplified this to a pass/fail model for the online environment. Previous assessments were so heavily informed by the clinical encounter that, unfortunately, they became irrelevant to provide any meaningful feedback to the student. Therefore, new clerkship summative assessment forms were created to better capture the student’s engagement in the course and provide feedback on their strengths and opportunities for growth (Appendix 3). This also better served to capture the student’s effort and fitness for residency in creation of the Medical School Performance Evaluation letter narratives and faculty letters of recommendation.

For EM-bound applicants, this course served to enhance their medical knowledge foundation in the field and enabled them to interact with the EM faculty in a virtual setting and avoid interruptions in their education from having a cancelled rotation. At our institution, these students were able to complete a home clinical rotation as soon as possible, allowing time for faculty to write a Standardized Letter of Evaluation and provide clinical feedback to the students. An interesting observation by the instructors who evaluated the group of EM-bound students involved in both the virtual and a subsequent traditional in-person clinical rotation was that those students who excelled in the virtual environment as evidenced by high test scores and good participation during online sessions, although they still did very well, did not necessarily maintain their top spots when evaluated during the clinical experience.

Alternatively, some students who were assessed as having average medical knowledge during the virtual clerkship, excelled in the clinical setting as they were able to showcase professional traits such as tenacity and ability to establish rapport, which are essential to the practice of EM. It is for this reason the virtual clerkship experience is not sufficient as the only method of exposure to the specialty for the EM-bound student. For those interested in EM as a career, we recommend that the student participate in an in-person clinical experience prior to applying to the specialty so that they can make an informed decision about whether EM is the right specialty choice for them.

It was our hope that the virtual clerkship would provide the same level and quality of conventional instructional resources needed to fulfill the learning needs of the fourth-year medical student. To assess the extent to which these aims were achieved, we designed a multimethod evaluation study that included the analysis of student scores on the EM shelf exam and a thematic study of comments elicited from a student focus group.

We performed a *t*-test for independent means comparing the composite scores of students completing the virtual rotation with the composite scores of students who completed the traditional rotation prior to the mandated shutdown of clinical education. Scores among the 56 students who completed the virtual rotation (X̄ = 81.18, standard deviation [SD] 6.55, 95% confidence interval [CI], 79.42, 82.93) were not statistically different from the 48 students completing the traditional rotation during that same academic year in the six months prior to the COVID-19 pandemic (X̄ = 79.38, SD 6.85, 95% CI, 77.39, 81.36), *t*(102) = 1.317, *P* = .174, 95% CI [−.808, 4.415]. This finding provides evidence that the development of students’ fund of knowledge in EM was not attenuated by their participation in the virtual rotation experience.

Post-course, we assembled a student focus group and asked participants to describe their thoughts about the merit, value, or shortcomings of the virtual clerkship. The following themes emerged from their comments:

### The virtual clerkship was well constructed and organized

- “Every week there was a theme, you know, like trauma, abdominal pain, or chest pain. Solid, worthwhile topics that were laid out for us.”
- “This was my favorite virtual thing that I’ve done. The clerkship director did a great job with providing us with a variety of cases and ways to learn.”
- “The other virtual clerkships I did allowed us to use whatever resources we wanted. That made things scattered and unorganized.”
- “There were daily assignments, reading, and cases we had to do. And there was accountability because you had to turn things in through Teams.”

### Quality resources and activities are paramount for authentic learning

- “I thought the podcasts were really great. She [the clerkship director] should continue to use them even after things go back to normal.”
- “Students got to choose a topic relevant to EM to make a presentation on. As a non-EM-bound student, getting to choose what to learn about was a very good approach as far as being valuable to me as a learner.”
- “She gave us so many high-quality EM resources. Anyone who does well with self-directed learning would benefit from this format.”

### Shortcomings of a virtual clerkship experience and suggestions for improvement

- “I miss the opportunity to interact with peers.”
- “Maybe try to have more small group cases and get more resident involvement. It is amazing to learn from near-peers.”
- “It is difficult to get audience interaction in the virtual setting. Have the instructors call on people randomly to ask questions and generate talk.”

## CONCLUSION

Students participating in this virtual clerkship in emergency medicine demonstrated a fund of knowledge at the course conclusion that did not differ from that of their peers who completed a traditional rotation in the specialty. Student comments indicate that this particular virtual clerkship was successful in meeting the learning needs of fourth-year medical students as a result of its design, organization, and use of quality learning resources. Opportunities for improvement of this experience are consistent with assessments of other virtual learning activities, namely, limitations in peer interaction and group learning dynamics.

## Supplementary Information







## Figures and Tables

**Table 1:** Objectives, instructional methods, and assessment strategies for a virtual emergency medicine clerkship curriculum.

Learning objective	Instructional design and means of assessment	Comparison to traditional in-person clerkship
Understand the complaint-directed history and physical exam	Role-playing using mock oral board format	Substitution of patients for residents and attendings during role-playing patient encounters
Develop a case-specific differential diagnosis	Team-based learning using real-life and textbook-based case presentations Virtual platform (Online MedEd CaseX) with immersive and interactive case play to allow learners to develop their differential after seeing video of patient and seeing the history and exam details. Role-playing using mock oral board format	Decreased opportunities for practice with lack of patient encounters
Present cases in a clear and concise fashion	Role-playing using mock oral board format	Decreased opportunities for practice with lack of patient encounters
Demonstrate an understanding of the use and interpretation of commonly ordered diagnostic studies	Team-based learning using real-life and textbook-based case presentations Role-playing using mock oral board format Virtual platform (Online MedEd CaseX) with immersive and interactive case play to allow learners to think about what they would order after seeing video of patient and seeing the history and exam details	Missed opportunity to practice writing real orders in the electronic health record
Develop appropriate case management plans	Team-based learning using real-life and textbook-based case presentations Role-playing using mock oral board format Virtual platform (Online MedEd CaseX) with immersive and interactive case play to allow learners to think about what they would order after seeing video of patient and seeing the history and exam details	Decreased opportunities for practice with lack of patient encounters
Demonstrate an adequate fund of knowledge	Traditional lectures, grand rounds, supplemental readings, podcasts Assessed with daily quizzes (formative) and the National Board of Medical Examiners Emergency Medicine Clerkship Shelf Exam (summative)	Able to go into depth and cover more topics formally than during the traditional model
Demonstrate understanding of indications, contraindication and techniques of basic procedural skills	Team-based learning using real-life and textbook-based case presentations	Unable to have the student demonstrate proficiency in the procedural skill itself
Demonstrate emergency recognition and management	Team-based learning using real-life and textbook-based case presentations Role-playing using mock oral board format	Substitution of patients for residents and attendings during role-playing patient encounters

**Table 2:** Virtual platforms with advantages and disadvantages.*

Resources	Advantages	Disadvantages
Microsoft Teams	Allows for the development of a virtual classroom, one platform to build and house the assignments and quizzes and to track grades Secure Easy file- and calendar-sharing; able to copy classroom for new rotation of learners without having to reload all the content and assignments	During the clerkship we noted difficulty with access for those not using their internal, institutional email address
Online MedEd Case X	Video of real patients captured by EMS or clinical staff of the patient interview	Not EM specific; cases were pulled from other clerkship content
	Interactive, allowing the learner to sequentially go through the history and physical exam, differential diagnosis, and treatment choices	Paid subscription
EM:RAP C3 series	EM-specific case series incorporating audio from patient encounters in the ED setting with commentary from emergency physicians	No visual components to allow learner to see patient Paid subscription
SAEM EM Curriculum	Video content for medical students on delivering an effective oral presentation, transferring care of a patient, and calling a consultation Excellent summary articles for supplemental reading	Asynchronous content; not interactive
Sublux Radiology App	Plain film radiology with anatomy and pathology overlays of imaging Includes normal radiographs for comparison Interactive for the learner, with clinical pearls and management of findings Free and available on iOS and Android platforms	
A Night in the ER App	Simulates reading CT images, including scrolling as on PACS imaging systems EM specific Interactive and labeled to highlight pathology Free	Available only on iOS; not available for Android

* This table offers the virtual platforms used in our virtual clerkship and is not meant to be a comprehensive list of all available platforms for online emergency medicine education.

*EMS*, emergency medical services; *EM:RAP*, Emergency Medicine Reviews and Perspectives; *SAEM*, Society for Academic Emergency Medicine; *EM*, emergency medicine; *CT*, computed tomography; *PACS*, picture archive and communication system.

**Table 3:** Challenges with virtual learning environments and proposed solutions.

Challenges identified	Proposed solutions
Lack of immersive experiential learning from direct patient contact	Include opportunities for interactive live didactic sessions, and employ multi-modal sessions including video, audio, team-based learning, role-playing, and group discussion
Limited opportunities for direct feedback to learners	Incorporate opportunities for mock oral board-style cases with time for debriefing and feedback
Lack of procedural skills training	Consider sending supplies, such as suture material, to learners to have them practice at home while watching procedure videos or during live didactic sessions
Low participation from learners during live sessions	Encourage students to have their cameras on when speaking during live interactive sessions Set expectations for attendance, participation and communication, and consider incorporation of expectations into institutional policy Incorporate methods such as games, polls, quizzes or breakout rooms, which have been shown to encourage student participation
