# Supplementary material for: Virtual Emergency Medicine Clerkship Curriculum during the COVID-19 Pandemic: Development, Application, and Outcomes
Source: West J Emerg Med. 2021 Apr 28;22(3):792–8. doi: 10.5811/westjem.2021.2.48430 (PMC8202996; doi:10.5811/westjem.2021.2.48430)
Supplement: Supplementary file 1 [file wjem-22-792-s001.docx]

**Advanced Emergency Medicine Clerkship (Virtual)**

|  |  |
| --- | --- |

**COURSE DESCRIPTION:**

This is a four-week course for the 4^th^ year medical student that combines independent, self-directed learning with live, synchronous team-based discussions that serves as a replacement to hands-on, patient care-oriented EM clerkship.

It is curated content built around the idea of “Simulated Patient Encounters” utilizing Online MedEd Case X videos of emergency medicine patients. It is organized by the most common chief complaints and procedures students will encounter in the clinical setting. Woven throughout the course are sessions in radiology and ECG interpretation and supplemental learning from textbooks, primary journal articles, podcasts, online board review, and FOAM blog posts.

The students complete a daily quiz to reinforce topics and in preparation for the shelf exam. Each student is also asked to prepare a short case presentation.

It utilizes the Microsoft Teams platform to build the assignments, quizzes and track grades. Online MedEd Case X and EM:RAP are proprietary resources utilized here for which our library has provided access to our students. The other resources are available free online or via download.

**OBJECTIVES:**

The Emergency Medicine rotation combines facets of all subspecialties while focusing on acute care management and critical care, improving differential diagnosis insight and skills, and coordinating inpatient and outpatient healthcare with primary care providers and other services.

1. Understand the complaint-directed H&PE
2. Develop a case-specific differential diagnosis
3. Present cases in a clear and concise fashion
4. Demonstrate an understanding of the use and interpretation of commonly ordered diagnostic studies
5. Develop appropriate case management plans
6. Demonstrate an adequate fund of knowledge
7. Demonstrate understanding of indications, contraindication and techniques of basic procedural skills
8. Demonstrate Emergency recognition and management

**REQUIRED TEXTS AND RESOURCES**:

1. Wolfson, A. B., Cloutier, R. L., Hendey, G. W., Ling, L., Rosen, C. L., & Schaider, J.

(2015). Harwood-Nuss clinical practice of emergency medicine. Philadelphia: Wolters Kluwer.

1. Rosh, A. J. (2012). Emergency medicine: pretest ; self-assessment and review. New York: McGraw-Hill Professional.
2. SAEM EM Curriculum - https://www.saem.org/cdem/education/online-education/m4-curriculum
3. EM:Rap.org
4. Online MedEd Case X - https://onlinemeded.org/spa/case-x
5. Sublux Radiology App, available for download on iOS device
6. A Night in the ER App, Available for download on iOS ipad device

**ATTENDANCE:**

Success in this course is dependent on active participation and engagement throughout the course. As such, students are required to complete all assignments by the due date, and to actively participate in class discussions.

**COMMUNICATION:**

Communications will be sent via email and also be posted in the Microsoft Teams Classroom.

**GRADING:**

Will be pass/ fail based on performance on the NBME shelf exam. Additionally, students will need to have completed the other assigned tasks.

**ASSIGNMENT SUBMISSION:**

Assignments are submitted via Microsoft Teams.

**COURSE REQUIREMENTS**

Deadlines for assignments are listed in the course calendar in Microsoft Teams

**Quiz #1 Introduction to Emergency Medicine .............................................................................. 5 points**

**Quiz #2 Chest Pain .......................................................................................................................... 5 points**

**Quiz #3 Shortness of Breath ........................................................................................................... 5 points**

**Quiz #4 Syncope ............................................................................................................................... 5 points**

**Quiz #5 EMS and Disaster Medicine ............................................................................................. 5 points**

**Quiz #6 Abdominal Pain ................................................................................................................. 5 points**

**Quiz #7 Trauma ............................................................................................................................... 5 points**

**Quiz #8 Neurologic Disorders ......................................................................................................... 5 points**

# Quiz #9 Psychiatry and Toxicology ................................................................................................ 5 points

**Quiz #10 Sepsis and Shock .............................................................................................................. 5 points**

**Quiz #11 Genitourinary Disorders ................................................................................................. 5 points**

**Quiz #12 Pediatric Fever and Illness ............................................................................................. 5 points**

**Quiz #13 Procedural Skills .............................................................................................................. 5 points**

**Mid-clerkship Exam ...................................................................................................................... 50 points**

**Case Presentation ........................................................................................................................... 25 points**

**Course Participation / Professionalism ........................................................................................ 25 points**

# NBME Shelf Exam ........................................................................................................................ 100 points

# Total 265 points
