# Supplementary material for: Virtual Emergency Medicine Clerkship Curriculum during the COVID-19 Pandemic: Development, Application, and Outcomes
Source: West J Emerg Med. 2021 Apr 28;22(3):792–8. doi: 10.5811/westjem.2021.2.48430 (PMC8202996; doi:10.5811/westjem.2021.2.48430)
Supplement: Supplementary file 2 [file wjem-22-792-s002.docx]

| **VIRTUAL EMERGENCY MEDICINE CLERKSHIP COURSE CALENDAR** | | | | | |
| --- | --- | --- | --- | --- | --- |
| **Day** | **Unit Topic** | **Activity** | **Instructions** | **Resources / Reference Material Provided** | **Assessment** |
| 1 | Introduction to Emergency Medicine (SAEM Curriculum Online)^1^ | - Medical Student Presentations in the Emergency Department^2^ | - Please read the following - Please watch the following video: | - Presenting in the ER^2^ - 3 minute Emergency Medicine Student Presentation.pdf^3^ | - Quiz #1: Introduction to Emergency Medicine |
|  |  | - Transfer of Care - Improving Handoffs in the Emergency Department | - Please watch the following video: <https://vimeo.com/223450018>^4^ | - Improving Patient Handoffs - Annals^5^ - Changes in Medical Errors after Implementation of a Handoff Program NEJM^6^ - Communication Gibson^7^ - Adapting the I-PASS Handoff Program for Emergency Department Inter-Shift Handoffs WJEM^8^ |  |
|  |  | - Stabilization of the Acutely Ill | - Please read the following | - Stabilization of the Acutely Ill Patient^9^ |  |
|  |  | - Approach to the Undifferentiated Patient | - Please read the following | - Approach to the Undifferentiated Patient^10^ |  |
|  |  | - Effective Consultation in the Emergency Department | - Go to the following link and watch: <https://vimeo.com/299218325>^11^      - Please read the following articles attached: | - Enhancing Communication to Improve Patient Safety and to Increase Patient Satisfaction.^12^      - The 5Cs of Consultation: Training Medical Students to Communicate Effectively in the Emergency Department^13^ - A prospective, randomized, controlled study demonstrating a novel, effective model of transfer of care between physicians: the 5 Cs of consultation. ^14^ |  |
| ORIENTATION SESSION LIVE | | - Web Based Microsoft Teams Meetings | - Join and Participate in Microsoft Teams Meeting |  |  |
| 2 | Chest Pain | - Simulated EM Shift: Virtual Patient Encounters | Please log onto <https://onlinemeded.org/spa/case-x>  Work through the following cases today:   - CARDIOLOGY 1 - "CC: tearing chest pain"^15^ - CARDIOLOGY 2 - "CC: palpitations"^16^ - CARDIOLOGY 3 - "CC: chest pain"^17^ | - Approach to Chest Pain (SAEM)^18^ | - Quiz #2 Chest Pain (Pre-Test EM)^19^ |
|  |  | - Simulated EM Shift: Podcast Case Presentations | - EM:Rap C3 Project March 2017 Chest Pain^20^ | - Mp3 files - Pdf summary - The HEART score for the assessment of patients with chest pain in the emergency department: A multinational validation study^21^ - Diagnostic pathways in acute pulmonary embolism: Recommendations of the PIOPED II pdf^22^ |  |
|  |  | - Killer EKG Patterns | Go to the following site: <https://litfl.com/killer-ecg-patterns/>^23^  Read the material. | - Killer EKG Patterns.png^23^ |  |
| 3 | Shortness of Breath | - Simulated EM Shift: Virtual Patient Encounters | Please log onto <https://onlinemeded.org/spa/case-x>  Work through the following cases today:   - PULMONOLOGY 1 - CC: Acute Shortness of Breath^24^ - PULMONOLOGY 2 - CC: Shortness of Breath^25^ - PULMONOLOGY 3 - CC: Shortness of Breath and cough^26^ - PULMONOLOGY 5 - CC: Shortness of Breath^27^ | - Approach to Shortness of Breath (SAEM)^28^ | - Quiz #3 Shortness of Breath (Pre-Test EM)^19^ |
|  |  | - Simulated EM Shift: Podcast Case Presentations | - EM:Rap C3 Project Dyspnea^29,30^ | - Mp3 files - Pdf summary |  |
|  |  | - Radiology Review: Chest XRays | Download the Sublux app onto your iOS device^31^   - Review the X-Ray findings under "Chest" | - Chest Radiograph SAEM^32^ |  |
| 4 | Syncope | - Simulated EM Shift: Virtual Patient Encounters | Please log onto <https://onlinemeded.org/spa/case-x>  Work through the following cases today:   - CARDIOLOGY 5: Witnessed Loss of Consciousness^33^ - ENDOCRINE 3: Found down with elevated glucose^34^ - INFECTIOUS DISEASE 2: Loss of Consciousness^35^ |  | - Quiz #4 Syncope (Pre-Test EM)^19^ |
|  |  | - Simulated EM Shift: Podcast Case Presentations | - EM:Rap C3 Project November 2016 Syncope^36^ | - Mp3 files - Pdf summary |  |
|  |  | - ECG Red Flags for Syncope and Pre-Syncope | Please watch the following videos:   - ECG Red Flags for Syncope and Pre-Syncope ^37^ <https://www.youtube.com/watch?v=raTTYV7_AsI> - Fainting Dead Away – Cardiogenic Syncope^38^ <https://www.youtube.com/watch?v=t1KuAtx7gmE> |  |  |
| WEEKLY CONFERENCE AND DIDACTICS #1 | | - Microsoft Teams Meeting | - Join scheduled online didactic session and participate in case discussions | - Powerpoint, Chest Pain, Shortness of Breath and Syncope |  |
| 5 | EMS and Disaster Response | - EMS and Disaster Response | - EM:Rap Crunch Time - EMS^39^ | - Mp4 file - EMS summary pdf | - Quiz #5 EMS (Pre-Test)^19^ |
|  |  | - Coronavirus for Medical Students | COVID-19 Resource Hub for AAMC^40^ <https://www.aamc.org/coronavirus-resources-academic-medicine-community> Personal Protective Equipment and Covid-19 | - EMRAP COVID-19 Response^41^ - PPE and COVID-19 article and video from NEJM^42^ |  |
| 6 | Abdominal Pain | - Simulated EM Shift: Virtual Patient Encounters | Please log onto <https://onlinemeded.org/spa/case-x>  Work through the following cases today:   - Gastroenterology 1: CC: Abdominal Pain^43^ - Gastroenterology 3: CC: Abdominal Pain^44^ - Gastroenterology 5: CC: Dark Tarry Stools^45^ - Surgery 2: Pediatric Abdominal Pain^46^ | - Approach to Abdominal Pain (SAEM)^47^ | - Quiz #6 Abdominal Pain (Pre-Test EM)^19^r |
|  |  | - Simulated EM Shift: Podcast Case Presentations | - EM:Rap C3 Pediatric Abdominal Pain February 2017^48^ - EM:Rap C3 Elderly Abdominal Pain January 2017^49^ | - Mp3 files - Pdf summary |  |
|  |  | - Radiology Review: Abdominal Pain | - Watch the attached videos on POCUS for AAA and Aortic Dissection (EM:RAP) - Open Sublux and review the Abdominal Films^31^ - Download and open "A Night in the ER" on iOS iPad to be able to scroll through CT imaging of the abdomen and pelvis^50,51^ | - Ultrasound of Aortic Aneursym^52^ - Ruptured AAA US^53^ - Aortic Dissection US^54^ - Read – Aortic Bedside US^55^ |  |
| 7 | Trauma | - Simulated EM Shift: Virtual Patient Encounters | Please log onto <https://onlinemeded.org/spa/case-x>  Work through the following cases today:   - Surgery 8 CC: Loss of consciousness, Chainsaw to knee^56^ - Surgery 12 CC: MVC, Shoulder Pain^57^ | - Approach to Trauma (SAEM)^58^ | - Quiz #7 Trauma (Pre-Test EM)^19^ |
|  |  | - Supplemental Case Study Reading | - Please read the attached files from SAEM | - Closed Head Injury^59^ - Orthopedic Injuries - Chest Trauma^60^ - FAST exam^61^ - Cervical Spine Imaging in Trauma^62^ |  |
| WEEKLY CONFERENCE AND DIDACTICS #2 | | - Microsoft Teams Meeting | - Join scheduled online didactic session and participate in case discussions | - Powerpoint, Abdominal Pain and Trauma – AAA and FAST exams |  |
| 8 | Neurologic Symptoms | - Simulated EM Shift: Virtual Patient Encounters | Please log onto <https://onlinemeded.org/spa/case-x>  Work through the following cases today:   - Neurology 1^63^ - Neurology 3^64^ - Neurology 5^65^ | - Approach to Headache (SAEM)^66^ - Approach to Ischemic Stroke (SAEM)^67^ | - Quiz #8 Neurologic Symptoms (Pre-Test EM)^19^ |
|  |  | - Simulated EM Shift: Podcast Case Presentations | - EM:Rap C3 Stroke podcast^68^ | - Mp3 files (Introduction. Ischemic, Hemorrhagic, Mimics, Disposition) |  |
|  |  | - Radiology: Brain Imaging | - Open “A Night In the ER” App on your iOS i-pad and review CT Images of the Head^50,51^   Watch the following video: How to Read CT of the Head^69^ <https://www.youtube.com/watch?v=MlRsfpb40pA>   - Read the attached document | - Brain Imaging (SAEM)^70^ - Reading Head CT: A Primer ^71^ |  |
| Midclerkship Examination | | SAEM M4 Exam^72^ | | | |
| 9 | Toxicology / Psychiatry | - Simulated EM Shift: Virtual Patient Encounters | Please log onto <https://onlinemeded.org/spa/case-x>  Work through the following cases today:   - Psychiatry 2 CC: Odd Behavior^73^ - Psychiatry 3 CC: Paranoia^74^ - Psychiatry 4 CC: Hyperventilation^75^ |  | - Quiz #9 Toxicology and Psychiatry (Pre-Test EM)^19^ |
|  |  | - Simulated EM Shift: Podcast Case Presentations - Psych | - EM:Rap C3 Psychiatric Emergencies 2019^76,77^ | - Mp3 files (Introduction, New Onset Psychosis, Delirium and Dementia, Acute Agitation) |  |
|  |  | - Simulated EM Shift: Podcast Case Presentations - Tox | - EM:Rap Crunch Time Toxicology^78^ | - Mp3 files (General Concepts, Agent/Antidote Game) - Approach to Poisonings pdf - Tox.pdf |  |
|  |  | - One Pill Can Kill: Pediatric Poisoning | - Read the following articles | - One Pill Can Kill Pediatric Poisoning^79^ - Approach to Poisonings^80^ - ACEP One Pill (or Sip) Can Kill Table^81^ |  |
|  |  | - The Toxic EKG | Watch the following video:   - Cardiovascular Toxicity in the Poisoned Patient^82^   <https://litfl.com/12-lead-ecg-in-toxicology/> | - https://vimeo.com/226552158^82^ |  |
| 10 | Shock and Sepsis | - Simulated EM Shift: Virtual Patient Encounters | Please log onto <https://onlinemeded.org/spa/case-x>  Work through the following cases today:   - Infectious Disease 1^83^ - Infectious Disease 3^84^ | - Sepsis pdf (SAEM)^85^ - Shock pdf (SAEM)^86^ | - Quiz #10 Shock (Pre-Test EM)^19^ |
|  |  | - Supplemental Material on Sepsis and Shock | Watch the following videos:   - <https://www.youtube.com/watch?v=4U0K1qVcLlA&t=226s> - <https://www.youtube.com/watch?v=CjCxumH3HLA> | - 5 Common Errors in Shock, Amal Mattu^87^ - The Crashing Patient^88^ |  |
| WEEKLY CONFERENCE AND DIDACTICS #3 | | - Microsoft Teams Meeting | - Join scheduled online didactic session and participate in case discussions | - Powerpoint Shock, Sepsis |  |
| 11 | GU Cases | - Simulated EM Shift: Podcast Case | - EM:Rap Crunch Time OB^89^ | - Mp3 files (Abnormal Uterine Bleeding, Miscarriage, Ovarian Torsion, Ectopic Pregnancy, Sexual Assault) | - Quiz #11 GU (Pre-Test)^19^ |
|  |  | - Simulated EM Shift: Virtual Patient Encounters | Log Into <https://onlinemeded.org/spa/cases>    Complete the following modules:   1. OB/GYN 1 CC: Pelvic Pain^90^ 2. Surgery 5 CC: Scrotal pain^91^ | - Ectopic Pregnancy (SAEM)^92^ - PID and TOA^93^ - Ovarian Torsion^94^ - Testicular Torsion^95^ |  |
| 12 | Pediatrics | - Simulated EM Shift: Virtual Patient Encounters | Please log onto <https://onlinemeded.org/spa/case-x>  Work through the following cases today:   - Pediatrics 1: Fever and cough^96^ - Pediatrics 2: Fever and Seizure^97^ | - Crying Child^98^ - Child Abuse (Non-accidental Trauma)^99^ - Nursemaid’s Elbow^100^ | - Quiz #12 Pediatrics (Pre-Test EM)^19^ |
|  |  | - Managing Procedural Anxiety in Children | - Read the following article and watch the video | - Managing Procedural Anxiety in Children^101^ |  |
|  |  | - Simulated EM Shift: Podcast Case Presentations – Pediatric Fever | - EM:Rap C3 Pediatric Fever^102^ | - Mp3 files (Introduction, General Approach, Pathogens, Summary) - Pediatric Fever (SAEM)^103^ |  |
| 13 | Procedural Skills | - Suture Lab | - Please watch the following videos | - EMHD Simple Interrupted^104^ - Forearm Laceration Repair^105^ - Scalp Laceration Repair with Staples^106^ - Comparison of Suture Materials^107^ - Deep Dermal Sutures^108^ - Corner Suture^109^ - Laceration Repair with Tape and Glue^110^ | Quiz #13 Procedural Skills (Pre-Test EM)^19^ |
|  |  | - Vascular Access | - Please watch the following videos | - Intraosseous access^111,112^ - Central Line Placement^113^ - Ultrasound Guided Peripheral IV Access^114^ |  |
|  |  | - Airway Management | - Please watch the following videos | - Bag Valve Mask Ventilation^115^ - Oral-Tracheal Intubation^116^ - C3: Airway Basics^117,118^ |  |
| WEEKLY CONFERENCE AND DIDACTICS #4 | | - Microsoft Teams Meeting | - Join scheduled online didactic session and participate in case discussions | - Student Assigned Presentations |  |
| FINAL ASSESSMENT | | NBME Shelf Exam | | | |

References

1. M4 Curriculum. https://www.saem.org/cdem/education/online-education/m4-curriculum. Accessed May 26, 2020.

2. Medical Student Presentations Video. https://www.saem.org/cdem/education/online-education/medical-student-presentations-video. Accessed May 26, 2020.

3. Davenport C, Honigman B, Druck J. The 3-Minute Emergency Medicine Medical Student Presentation: A Variation on a Theme. *Acad Emerg Med*. 2008;15(7):683-687. doi:10.1111/j.1553-2712.2008.00145.x

4. Transfer of Care Video. https://www.saem.org/cdem/education/online-education/transfer-of-care-video. Accessed May 26, 2020.

5. Cheung DS, Kelly JJ, Beach C, et al. PATIENT SAFETY/CONCEPTS Improving Handoffs in the Emergency Department. 2009. doi:10.1016/j.annemergmed.2009.07.016

6. Starmer AJ, Spector ND, Srivastava R, et al. Changes in Medical Errors after Implementation of a Handoff Program. *N Engl J Med*. 2014;371(19):1803-1812. doi:10.1056/NEJMsa1405556

7. Gibson SC, Ham JJ, Apker J, Mallak LA, Johnson NA. Communication, Communication, Communication: The Art of the Handoff. *Ann Emerg Med*. 2010;55:181-183. doi:10.1016/j.annemergmed.2009.10.009

8. Heilman JA, Flanigan M, Nelson A, Johnson T, Yarris LM. Adapting the I-PASS handoff program for emergency department inter-shift handoffs. *West J Emerg Med*. 2016;17(6):756-761. doi:10.5811/westjem.2016.9.30574

9. Stabilization of the Acutely Ill Patient. https://www.saem.org/cdem/education/online-education/m3-curriculum/group-stabilization-of-the-acutely-ill-patient/stabilization-of-the-acutely-ill-patient. Accessed May 26, 2020.

10. Approach to the Undifferentiated Patient. https://www.saem.org/cdem/education/online-education/m3-curriculum/group-approach-to-the-undifferentiated-patient/approach-to-the-undifferentiated-patient. Accessed May 26, 2020.

11. Effective Consultation in Emergency Medicine Video. https://www.saem.org/cdem/education/online-education/effective-consultation-in-emergency-medicine-video. Accessed May 26, 2020.

12. Burgener AM. Enhancing Communication to Improve Patient Safety and to Increase Patient Satisfaction. *Health Care Manag (Frederick)*. 2017;36(3):238-243. doi:10.1097/HCM.0000000000000165

13. Kessler CS, Tadisina KK, Saks M, et al. The 5Cs of Consultation: Training Medical Students to Communicate Effectively in the Emergency Department. *J Emerg Med*. 2015;49(5):713-721. doi:10.1016/j.jemermed.2015.05.012

14. Kessler CS, Afshar Y, Sardar G, Yudkowsky R, Ankel F, Schwartz A. A Prospective, Randomized, Controlled Study Demonstrating a Novel, Effective Model of Transfer of Care between Physicians: The 5 Cs of Consultation. *Acad Emerg Med*. 2012;19(8):968-974. doi:10.1111/j.1553-2712.2012.01412.x

15. Case X - Cardiology 1. https://onlinemeded.org/spa/cases/cardiology-1. Accessed May 26, 2020.

16. Case X - Cardiology 2. https://onlinemeded.org/spa/cases/cardiology-2. Accessed May 26, 2020.

17. Case X - Cardiology 3. https://onlinemeded.org/spa/cases/cardiology-3. Accessed May 26, 2020.

18. Chest Pain. https://www.saem.org/cdem/education/online-education/m3-curriculum/group-focused-chief-complaint-history-physical-examination-and-differential-diagnosis/chest-pain. Accessed May 26, 2020.

19. Rosh AJ. *Emergency Medicine: Pretest Self-Assessment and Review*. Second. New York, NY: McGraw Hill Medical; 2009.

20. C3 - C3 - Chest Pain - Introduction | EM:RAP. https://www.emrap.org/episode/c3chestpain/c3chestpain. Accessed May 26, 2020.

21. A.J. S, L. C, B.E. B, et al. The HEART score for the assessment of patients with chest pain in the emergency department: A multinational validation study. *Crit Pathw Cardiol*. 2013;12(3):121-126. doi:10.1097/HPC.0b013e31828b327e LK - http://resolver.ebscohost.com/openurl?sid=EMBASE&issn=1535282X&id=doi:10.1097%2FHPC.0b013e31828b327e&atitle=The+HEART+score+for+the+assessment+of+patients+with+chest+pain+in+the+emergency+department%3A+A+multinational+validation+study&stitle=Crit.+Pathways+Cardiol.&title=Critical+Pathways+in+Cardiology&volume=12&issue=3&spage=121&epage=126&aulast=Six&aufirst=A.+Jacob&auinit=A.J.&aufull=Six+A.J.&coden=CPCRC&isbn=&pages=121-126&date=2013&auinit1=A&auinitm=J

22. Stein PD, Woodard PK, Weg JG, et al. Diagnostic pathways in acute pulmonary embolism: Recommendations of the PIOPED II - Investigators. *Radiology*. 2007;242(1):15-21. doi:10.1148/radiol.2421060971

23. Killer ECG Patterns • LITFL Medical blog • ECG Library. https://litfl.com/killer-ecg-patterns/. Accessed May 26, 2020.

24. Case X - Pulmonology 1. https://onlinemeded.org/spa/cases/pulmonology-1. Accessed May 26, 2020.

25. Case X - Pulmonology 2. https://onlinemeded.org/spa/cases/pulmonology-2. Accessed May 26, 2020.

26. Case X - Pulmonology 3. https://onlinemeded.org/spa/cases/pulmonology-3. Accessed May 26, 2020.

27. Case X - Pulmonology 5. https://onlinemeded.org/spa/cases/pulmonology-5. Accessed May 26, 2020.

28. Shortness of Breath. https://www.saem.org/cdem/education/online-education/m4-curriculum/group-m4-approach-to/shortness-of-breath. Accessed May 26, 2020.

29. C3 - C3 - Dyspnea - Part1 | EM:RAP. https://www.emrap.org/episode/c3dyspneapart1/c3dyspneapart1. Accessed May 26, 2020.

30. C3 - C3 - Dyspnea Part II | EM:RAP. https://www.emrap.org/episode/c3dyspneapartii/c3dyspneapartii. Accessed May 26, 2020.

31. ‎Sublux on the App Store. https://apps.apple.com/us/app/sublux/id1332240487. Accessed May 26, 2020.

32. Chest Radiograph. https://www.saem.org/cdem/education/online-education/m3-curriculum/group-diagnostic-testing/radiographic-interpretation/chest-radiograph. Accessed May 26, 2020.

33. Case X - Cardiology 5. https://onlinemeded.org/spa/cases/cardiology-5. Accessed May 26, 2020.

34. Case X - Endocrine 3. https://onlinemeded.org/spa/cases/endocrine-3. Accessed May 26, 2020.

35. Case X - Infectious Disease 2. https://onlinemeded.org/spa/cases/infectious-disease-2. Accessed May 26, 2020.

36. C3 - Syncope - Introduction | EM:RAP. https://www.emrap.org/episode/c3syncope/syncope. Accessed May 26, 2020.

37. ECG Red Flags for Syncope and Pre-Syncope - YouTube. https://www.youtube.com/watch?v=raTTYV7_AsI. Accessed May 26, 2020.

38. Fainting Dead Away - Cardiogenic Syncope | The Heart Course - YouTube. https://www.youtube.com/watch?v=t1KuAtx7gmE. Accessed May 26, 2020.

39. Crunch Time - Introduction & Terminology | EM:RAP. https://www.emrap.org/episode/ems/introduction. Accessed May 26, 2020.

40. Coronavirus (COVID-19) Resource Hub | AAMC. https://www.aamc.org/coronavirus-covid-19-resource-hub. Accessed May 26, 2020.

41. COVID-19 RESPONSE. https://covid.emrap.org/. Accessed May 26, 2020.

42. Ortega R, Gonzalez M, Nozari A, Canelli R. Personal Protective Equipment and Covid-19. Ingelfinger JR, ed. *N Engl J Med*. May 2020:NEJMvcm2014809. doi:10.1056/NEJMvcm2014809

43. Case X - Gastroenterology 1. https://onlinemeded.org/spa/cases/gastroenterology-1. Accessed May 26, 2020.

44. Case X - Gastroenterology 3. https://onlinemeded.org/spa/cases/gastroenterology-3. Accessed May 26, 2020.

45. Case X - Gastroenterology 5. https://onlinemeded.org/spa/cases/gastroenterology-5. Accessed May 26, 2020.

46. Case X - Surgery 2. https://onlinemeded.org/spa/cases/surgery-2. Accessed May 26, 2020.

47. Abdominal Pain. https://www.saem.org/cdem/education/online-education/m4-curriculum/group-m4-approach-to/approach-to-abdominal-pain. Accessed May 26, 2020.

48. C3 - Introduction and Case | EM:RAP. https://www.emrap.org/episode/c3pediatric/introductionand. Accessed May 26, 2020.

49. C3 - Introduction - Abdominal Pain in the Elderly | EM:RAP. https://www.emrap.org/episode/c3elderly/introduction. Accessed May 26, 2020.

50. Drexler IR. One Night in the ED. *J Digit Imaging*. 2015;28(4):386-388. doi:10.1007/s10278-015-9797-1

51. ‎One Night in the ED en App Store. https://apps.apple.com/us/app/radiology-2-0-one-night-in-the-ed-ipad-version/id404368189?l=es. Accessed May 26, 2020.

52. HD - Ultrasound of Aortic Aneurysm | EM:RAP. https://www.emrap.org/episode/ultrasoundof6/ultrasoundof. Accessed May 26, 2020.

53. HD - Ruptured AAA | EM:RAP. https://www.emrap.org/episode/rupturedaaa/rupturedaaa1. Accessed May 26, 2020.

54. HD - Ultrasound of Aortic Dissection | EM:RAP. https://www.emrap.org/episode/ultrasoundof/ultrasoundof. Accessed May 26, 2020.

55. AAA exam. https://www.saem.org/cdem/education/online-education/m3-curriculum/bedside-ultrasonagraphy/aaa-exam. Accessed May 26, 2020.

56. Case X - Surgery 8. https://onlinemeded.org/spa/cases/surgery-8. Accessed May 26, 2020.

57. Case X - Surgery 12. https://onlinemeded.org/spa/cases/surgery-12. Accessed May 26, 2020.

58. Boulger C, Ostro BM. *The Approach To Trauma Author Credentials*.

59. Closed Head Injury. https://www.saem.org/cdem/education/online-education/m4-curriculum/group-m4-trauma/closed-head-injury. Accessed May 26, 2020.

60. Chest Trauma. https://www.saem.org/cdem/education/online-education/m4-curriculum/group-m4-trauma/chest-trama. Accessed May 26, 2020.

61. FAST exam. https://www.saem.org/cdem/education/online-education/m3-curriculum/bedside-ultrasonagraphy/fast-exam. Accessed May 26, 2020.

62. Cervical Spine Imaging in Trauma. https://www.saem.org/cdem/education/online-education/m3-curriculum/group-traumatic-and-orthopedic-injuries/cervical-spine-imaging-in-trauma. Accessed May 26, 2020.

63. Case X - Neurology 1. https://onlinemeded.org/spa/cases/neurology-1. Accessed May 26, 2020.

64. Case X - Neurology 3. https://onlinemeded.org/spa/cases/neurology-3. Accessed May 26, 2020.

65. Case X - Neurology 5. https://onlinemeded.org/spa/cases/neurology-5. Accessed May 26, 2020.

66. Headache. https://www.saem.org/cdem/education/online-education/m4-curriculum/group-m4-approach-to/headache. Accessed May 26, 2020.

67. Ischemic Stroke. https://www.saem.org/cdem/education/online-education/m4-curriculum/group-m4-neurology/ischemic-stroke. Accessed May 26, 2020.

68. C3 - C3 - Stroke - Introduction | EM:RAP. https://www.emrap.org/episode/c32018january/c3stroke. Accessed May 26, 2020.

69. How to Read a CT Scan of the Head - MEDZCOOL - YouTube. https://www.youtube.com/watch?v=MlRsfpb40pA. Accessed May 26, 2020.

70. Brain Imaging. https://www.saem.org/cdem/education/online-education/m3-curriculum/group-diagnostic-testing/radiographic-interpretation/brain-imaging. Accessed May 26, 2020.

71. Broder JS. Head computed tomography interpretation in trauma: A primer. *Psychiatr Clin North Am*. 2010;33(4):821-854. doi:10.1016/j.psc.2010.08.006

72. Testing. https://www.saem.org/cdem/education/testing. Accessed May 26, 2020.

73. Case X - Psychiatry 2. https://onlinemeded.org/spa/cases/psychiatry-2. Accessed May 26, 2020.

74. Case X - Psychiatry 3. https://onlinemeded.org/spa/cases/psychiatry-3. Accessed May 26, 2020.

75. Case X - Psychiatry 4. https://onlinemeded.org/spa/cases/psychiatry-4. Accessed May 26, 2020.

76. C3 - C3 - Psychiatric Emergencies – Introduction | EM:RAP. https://www.emrap.org/episode/c3psychiatric/c3psychiatric. Accessed May 26, 2020.

77. C3 - Psychiatric Emergencies Part 2 - Introduction | EM:RAP. https://www.emrap.org/episode/c3psychiatric1/psychiatric. Accessed May 26, 2020.

78. Crunch Time - General Concepts Part 1 | EM:RAP. https://www.emrap.org/episode/tox/generalconcepts1. Accessed May 26, 2020.

79. emDOCs.net – Emergency Medicine EducationToxCard: Pediatric Toxicology - One Pill/Taste Can Kill - emDOCs.net - Emergency Medicine Education. http://www.emdocs.net/toxcard-pediatric-toxicology-one-pill-taste-can-kill/. Accessed May 26, 2020.

80. By W, Al’ai Alvarez : *Approach to Poisonings and Ingestions*.

81. ACEP // American College of Emergency Physicians. https://www.acep.org/how-we-serve/sections/toxicology/news/march-2016/one-pill-or-sip-can-kill/. Accessed May 26, 2020.

82. 12-lead ECG in Toxicology • LITFL • Toxicology Library. https://litfl.com/12-lead-ecg-in-toxicology/. Accessed May 26, 2020.

83. Case X - Infectious Disease 1. https://onlinemeded.org/spa/cases/infectious-disease-1. Accessed May 26, 2020.

84. Case X - Infectious Disease 3. https://onlinemeded.org/spa/cases/infectious-disease-3. Accessed May 26, 2020.

85. Sepsis. https://www.saem.org/cdem/education/online-education/m4-curriculum/group-m4-approach-to/sepsis. Accessed May 26, 2020.

86. Shock. https://www.saem.org/cdem/education/online-education/m4-curriculum/group-m4-approach-to/shock. Accessed May 26, 2020.

87. 5 Common Errors in Shock, Amal Mattu, Essentials of Emergency Medicine 2018 - YouTube. https://www.youtube.com/watch?v=4U0K1qVcLlA&t=226s. Accessed May 26, 2020.

88. The Crashing Patient - YouTube. https://www.youtube.com/watch?v=CjCxumH3HLA. Accessed May 26, 2020.

89. Crunch Time - Abnormal Uterine Bleeding | EM:RAP. https://www.emrap.org/episode/obgyn/abnormaluterine. Accessed May 26, 2020.

90. Case X - OB/Gyn 1. https://onlinemeded.org/spa/cases/ob-gyn-1. Accessed May 26, 2020.

91. Case X - Surgery 5. https://onlinemeded.org/spa/cases/surgery-5. Accessed May 26, 2020.

92. Ectopic Pregnancy - Genitourinary. https://www.saem.org/cdem/education/online-education/m4-curriculum/group-m4-genitourinary/ectopic-pregnancy---genitourinary. Accessed May 26, 2020.

93. PID + TOA. https://www.saem.org/cdem/education/online-education/m4-curriculum/group-m4-genitourinary/pid-toa. Accessed May 26, 2020.

94. Ovarian Torsion. https://www.saem.org/cdem/education/online-education/m4-curriculum/group-m4-genitourinary/ovarian-torsion. Accessed May 26, 2020.

95. Testicular Torsion. https://www.saem.org/cdem/education/online-education/m4-curriculum/group-m4-genitourinary/testicular-torsion. Accessed May 26, 2020.

96. Case X - Pediatrics 1. https://onlinemeded.org/spa/cases/pediatrics-1. Accessed May 26, 2020.

97. Case X - Pediatrics 2. https://onlinemeded.org/spa/cases/pediatrics-2. Accessed May 26, 2020.

98. Crying Child. https://www.saem.org/cdem/education/online-education/peds-em-curriculum/approach-to/crying-child. Accessed May 26, 2020.

99. Child Abuse (Non-accidental Trauma). https://www.saem.org/cdem/education/online-education/peds-em-curriculum/gu-ob/child-abuse-(non-accidental-trauma). Accessed May 26, 2020.

100. Nursemaid’s Elbow. https://www.saem.org/cdem/education/online-education/peds-em-curriculum/nursemaid’s-elbow#!#39;s-elbow. Accessed May 26, 2020.

101. Krauss BS, Krauss BA, Green SM. Managing Procedural Anxiety in Children. *N Engl J Med*. 2016;374(16):e19. doi:10.1056/NEJMvcm1411127

102. C3 - C3 - Pediatric Fever - Introduction | EM:RAP. https://www.emrap.org/episode/c3pediatric1/c3pediatric. Accessed May 26, 2020.

103. Fever. https://www.saem.org/cdem/education/online-education/peds-em-curriculum/approach-to/fever. Accessed May 26, 2020.

104. HD - Simple Interrupted Sutures | EM:RAP. https://www.emrap.org/episode/simple/simple. Accessed May 26, 2020.

105. HD - Forearm Laceration Repair | EM:RAP. https://www.emrap.org/episode/forearm/forearm. Accessed May 26, 2020.

106. HD - Scalp Laceration with Foreign Body | EM:RAP. https://www.emrap.org/episode/scalplaceration/scalplaceration. Accessed May 26, 2020.

107. HD - Comparison of Suture Materials | EM:RAP. https://www.emrap.org/episode/comparisonof/comparisonof. Accessed May 26, 2020.

108. HD - Deep Dermal Sutures | EM:RAP. https://www.emrap.org/episode/deepdermal/deepdermal. Accessed May 26, 2020.

109. HD - Corner Suture | EM:RAP. https://www.emrap.org/episode/cornersuture/cornersuture. Accessed May 26, 2020.

110. HD - Skin Tape and Glue for Wound Repair | EM:RAP. https://www.emrap.org/episode/skintapeandglue/skintapeandglue. Accessed May 26, 2020.

111. Dev SP, Stefan RA, Saun T, Lee S. Insertion of an Intraosseous Needle in Adults. *N Engl J Med*. 2014;370(24):e35. doi:10.1056/NEJMvcm1211371

112. Nagler J, Krauss B. Intraosseous catheter placement in children. *N Engl J Med*. 2011;364(8):e14(1). doi:10.1056/NEJMvcm0900916

113. Ortega R, Song M, Hansen CJ, Barash P. Ultrasound-guided internal jugular vein cannulation. *N Engl J Med*. 2010;362(16):e57(1). doi:10.1056/NEJMvcm0810156

114. Joing S, Strote S, Caroon L, et al. Ultrasound-Guided Peripheral IV Placement. *N Engl J Med*. 2012;366(25):e38. doi:10.1056/NEJMvcm1005951

115. Ortega R, Mehio AK, Woo A, Hafez DH. Positive-Pressure Ventilation with a Face Mask and a Bag-Valve Device. *N Engl J Med*. 2007;357(4):e4. doi:10.1056/NEJMvcm071298

116. Kabrhel C, Thomsen TW, Setnik GS, Walls RM. Orotracheal Intubation. *N Engl J Med*. 2007;356(17):e15. doi:10.1056/NEJMvcm063574

117. C3 - Airway Basics Part 1 - Introduction | EM:RAP. https://www.emrap.org/episode/c3airwaybasics/airwaybasics. Accessed May 26, 2020.

118. C3 - Airway Basics Part 2 - Preoxygenation | EM:RAP. https://www.emrap.org/episode/c3airwaybasics1/airwaybasics. Accessed May 26, 2020.
