# Supplementary material for: Virtual Emergency Medicine Clerkship Curriculum during the COVID-19 Pandemic: Development, Application, and Outcomes
Source: West J Emerg Med. 2021 Apr 28;22(3):792–8. doi: 10.5811/westjem.2021.2.48430 (PMC8202996; doi:10.5811/westjem.2021.2.48430)
Supplement: Supplementary file 3 [file wjem-22-792-s003.docx]

**Advanced Emergency Medicine Clerkship (Virtual)**

**Summative Assessment**

1. Participants in assessment of this student
2. Areas of Strength
3. Targets for Growth
4. Does the student pass this clerkship? Yes / No
5. How would you rate the student’s effort during this clerkship?

| Low | Modest | Adequate | More than Satisfactory | Exemplary |
| --- | --- | --- | --- | --- |

1. The student entered the elective with the fund of knowledge and/or skill on the topic I would expect a 4^th^ year medical student.

| Strongly Disagree | Disagree | Neither agree nor disagree | Agree | Strongly Agree |
| --- | --- | --- | --- | --- |

1. The student completed the elective with the fund of knowledge and/or skill on the topic I would expect a 4^th^ year medical student.

| Strongly Disagree | Disagree | Neither agree nor disagree | Agree | Strongly Agree |
| --- | --- | --- | --- | --- |

1. How often was the student fully prepared for the day’s learning activities during this elective?

| Practically never | Occasionally | About half the time | More often than not | Practically always |
| --- | --- | --- | --- | --- |

1. The student demonstrated an understanding of how to continue self-directed learning on the topics covered during the clerkship.

| Strongly Disagree | Disagree | Neither agree nor disagree | Agree | Strongly Agree |
| --- | --- | --- | --- | --- |

1. The student recognizes the limits of their knowledge or competency on this topic

| Strongly Disagree | Disagree | Neither agree nor disagree | Agree | Strongly Agree |
| --- | --- | --- | --- | --- |

1. How would you rate the student’s level of understanding of contemporary issues or current professional debate on the topic(s) covered during this clerkship?

| Poor/Fail | Below average | Satisfactory | Very Good | Superior |
| --- | --- | --- | --- | --- |

1. How would you characterize the student’s overall performance for the elective?

| Poor/Fail | Below average | Satisfactory | Very Good | Superior |
| --- | --- | --- | --- | --- |

1. The student is able to recognize normal and abnormal vital signs as they relate to patient- and disease-specific factors as potential etiologies of a patient's decompensation. (EPA-10)

| Fails to recognize trends or variations of vital signs in a decompensating patient | Demonstrates limited ability to gather, filter, prioritize, and connect pieces of information to form a patient specific differential diagnosis in an urgent or emergent setting | Progressing to next level but not consistently demonstrated | Recognizes outliers or unexpected results or data and seeks out an explanation | Progressing to next level but not consistently demonstrated | Recognizes variations of patient's vital signs based on patient- and disease- specific factor  Gathers, filters, and prioritizes information related to a patient's decompensation in an urgent or emergent setting |
| --- | --- | --- | --- | --- | --- |

1. Recognize severity of a patient's illness and indications for escalating care and initiate interventions and management

| Does not recognize change in patient's clinical status or seek help when a patient requires urgent or emergent care | Misses abnormalities in patient's clinical status or does not anticipate next steps  May be distracted by multiple problems or have difficulty prioritizing | Progressing toward next level but not yet consistent | Recognizes concerning clinical symptoms or unexpected results or data  Asks for help | Progressing toward next level but not yet consistent | Responds to early clinical deterioration and seeks timely help  Prioritizes patients who need immediate care and initiates critical interventions |
| --- | --- | --- | --- | --- | --- |

1. Please provide narrative feedback of the student’s performance to be included in the MSPE/Dean’s Letter.
